# Efficacy of three BCG strains (Connaught, TICE and RIVM) with or without secondary resection (re-TUR) for intermediate/high-risk non-muscle-invasive bladder cancers: results from a retrospective single-institution cohort analysis

**DOI:** 10.1007/s00432-021-03571-0

**Published:** 2021-03-06

**Authors:** Francesco Del Giudice, Gian Maria Busetto, Martin S. Gross, Martina Maggi, Alessandro Sciarra, Stefano Salciccia, Matteo Ferro, Isabella Sperduti, Simone Flammia, Vittorio Canale, Benjamin I. Chung, Simon L. Conti, Michael L. Eisenberg, Eila C. Skinner, Ettore De Berardinis

**Affiliations:** 1 Department of Maternal-Infant and Urological Sciences, Policlinico Umberto I Hospital, “Sapienza” University of Rome, Viale del Policlinico 155, 00161 Rome, Italy; 2 Department of Urology, Stanford Medical Center, Stanford, CA USA; 3 Section of Urology, Dartmouth-Hitchcock Medical Center, Lebanon, NH 03766 USA; 4 Department of Urology, European Institute of Oncology (IEO), IRCCS, Milan, Italy; 5 Biostatistical Unit, IRCCS, Regina Elena Hospital, Rome, Italy

**Keywords:** Bladder cancer, Re-TUR, BCG strain, BCG-Connaught, BCG-TICE, BCG-RIVM, Recurrence-free survival, Progression-free survival, Cancer-specific survival

## Abstract

**Purpose:**

(I) To evaluate the clinical efficacy of three different BCG strains in patients with intermediate-/high-risk non-muscle-invasive bladder cancer (NMIBC). (II) To determine the importance of performing routine secondary resection (re-TUR) in the setting of BCG maintenance protocol for the three strains.

**Methods:**

NMIBCs who received an adjuvant induction followed by a maintenance schedule of intravesical immunotherapy with BCG Connaught, TICE and RIVM. Only BCG-naïve and those treated with the same strain over the course of follow-up were included. Cox proportional hazards model was developed according to prognostic factors by the Spanish Urological Oncology Group (CUETO) as well as by adjusting for the implementation of re-TUR.

**Results:**

*n = *422 Ta-T1 patients (Connaught, *n = *146; TICE, *n = *112 and RIVM, *n = *164) with a median (IQR) follow-up of 72 (60–85) were reviewed. Re-TUR was associated with improved recurrence and progression outcomes (HR_RFS_: 0.63; 95% CI 0.46–0.86; HR_PFS_: 0.55; 95% CI 0.31–0.86). Adjusting for CUETO risk factors and re-TUR, BGC TICE and RIVM provided longer RFS compared to Connaught (HR_TICE_: 0.58, 95% CI 0.39–0.86; HR_RIVM_: 0.61, 95% CI 0.42–0.87) while no differences were identified between strains for PFS and CSS. Sub-analysis of only re-TUR cases (*n = *190, 45%) showed TICE the sole to achieve longer RFS compared to both Connaught and RIVM.

**Conclusion:**

Re-TUR was confirmed to ensure longer RFS and PFS in intermediate-/high-risk NMIBCs but did not influence the relative single BCG strain efficacy. When routinely performing re-TUR followed by a maintenance BCG schedule, TICE was superior to the other strains for RFS outcomes.

**Supplementary Information:**

The online version contains supplementary material available at 10.1007/s00432-021-03571-0.

## Introduction

Non-muscle invasive bladder cancers (NMIBCs) represent a heterogeneous category of tumors associated with high recurrence (30–80%) and progression (25–50%) rates, depending on the risk profile, and leading to cancer death after bladder-sparing treatment within 5 years in about 16–23% of cases (Ferlay et al. (2012); CompeÃÅrat et al. 2015). The highest risk subtype of NMIBC represented by high-grade T1 (HG T1) can reach an almost 40% rate of recurrence and 20% of progression at 5 years, despite adjuvant intravesical therapy with Bacillus Calmette–Guérin (BCG) (Bosch and Alfred 2011). From the initial introduction more than 35 years ago, BCG remains the gold standard organ-sparing option for patients classified as intermediate/high risk according to European Association of Urology (EAU) Guidelines (Babjuk et al. 2020; Witjes et al. 2020). Several series have demonstrated BCG’s superiority when compared with transurethral resection of bladder tumor (TURBT) alone or in combination with intravesical chemotherapy (Shelley et al. 2004; Han and Pan 2006; Malmström et al. 2009).

For optimal efficacy, BCG should be given in a maintenance schedule to prevent recurrence in patients with intermediate-/high-risk tumors, and evidence suggests that the three-year maintenance protocol originally described by Donald Lamm (2000) is more effective than 1-year (Lamm et al. 2000; Oddens et al. 2013). Despite this, many patients appropriate for intravesical immunotherapy will not complete the treatment protocol as planned due to side effects, efficacy, compliance and unfortunately shortage of BCG availability. The current worldwide BCG shortage has made the comparison of different strains, and potentially strain substitution, particularly of interest. To date, few studies have been conducted that compare strain efficacy, optimal dose and toxicity in the clinical setting with many limitations including variable maintenance schedules and BCG doses. Regarding induction protocol, a Dutch randomized clinical trial (RCT) (Vegt et al. 1995) observed RIVM to be superior in terms of recurrence-free survival (RFS) compared to TICE, and a second RCT in the same setting demonstrated Connaught to better prevent recurrence when compared to TICE (Rentsch et al. 2014). The largest European cohort of high-risk NMIBCs, published by Witjes et al. (2016), compared the 2 most widely used BCG strains, Connaught and TICE, and clearly revealed the independent importance of maintenance and how with this schedule TICE resulted in better recurrence-free outcomes.

An important frequently omitted variable among the available trials is information regarding re-staging procedures (re-TUR) and their potential influence on survival outcomes. In particular, for NMIBCs, residual tumor rates may vary between 33 and 76% for all cases, including 27–72% and 33–78% for Ta and T1 tumors respectively (Cumberbatch et al. 2018). Also, 7–30% of these patients are understaged after initial TURBT, increasing up to 45% when resection does not include detrusor muscle (Shindo et al. 2014). As a result, both intravesical chemotherapy and BCG do not appear to reliably compensate for inadequate resection and are not recommended to replace secondary resection, especially for the highest risk category of HG T1 (Mack et al. 2001; Herr 2005).

To explore this unresolved topic related to potential efficacy differences among BCG strains, we reviewed our historical cohort of NMIBC patients who were assigned to the most used BCG strains (Connaught, TICE and RIVM). The main objective of our analysis was to verify the existence of survival differences among these three stains, with a secondary goal of analyzing the weighted importance of secondary resection procedures in these patients.

## Patients and methods

After Institutional Review Board (IRB) approval, we performed a retrospective cohort study on intermediate-/high-risk BCG-naïve NMIBC patients who received induction followed by maintenance with three different BCG strains. Data were also collected on patients who underwent re-TUR within 2–8 weeks following primary resection.

Each participant enrolled in the study had signed an informed consent before undergoing intravesical BCG therapy according to the European Association of Urology (EAU) and Good Clinical Practice (GCP) Guidelines, and the ethical principles of the latest version of the Declaration of Helsinki.

Patients with muscle-invasive disease (≥ T2), upper tract urothelial cancer (UTUC), non-urothelial carcinoma, previous BCG, incomplete/missing data, or who did not initially receive BCG were excluded. The study design is summarized in Supplementary Fig. 1.

Recurrence was defined as tumor relapse in the bladder or prostatic urethra, regardless of tumor stage. Progression was defined as ≥ T2 tumor relapse in the bladder or prostatic urethra. The cause of death was determined from death certificates and chart review.

Full BCG consisted of induction and 7 maintenance courses. Six-week induction started 2–3 weeks after staging TURBT or re-TUR. Maintenance therapy was 3 weekly instillations every 3 months for the first two schedules and then every 6 months (Lamm 2000). Patients were only included if they were treated with the same BCG strain throughout follow-up. The three BCG strains were: BCG-Connaught (ImmuCyst®, Sanofi Pasteur, France) BCG-TICE (OncoTICE®, MSD, USA) BCG-RIVM (Medac®, D-20354, Germany). BCG choice was made on the basis of availability, price and supply.

### Statistical analysis

Pearson chi-square test or Fisher’s exact test measured the association between variables. Kruskal–Wallis test measured association among quantitative variables. Kaplan–Meier method tested univariate effect of BCG strain on survival outcomes. Log-rank test assessed subgroup differences adjusted for multiple comparison (Dunn–Sidak) when appropriate. For the three strains, study end points were recurrence- and progression-free survival (RFS, PFS), defined as months for any stage/grade to relapse (RFS) and months for rise to T2 or higher stage (PFS). An additional end point was cancer-specific survival (CSS).

Times to events were calculated by taking the date of starting BCG as time zero. Patients without an event were censored at the last follow-up. Cox proportional hazards multivariable regression analysis adjusted for the number of CUETO prognostic factors in BCG maintenance patients (Fernandez-Gomez et al. 2009). This model adjusted for age (< 60, 60–70 and ≥ 70 years), gender, prior recurrence, tumor number (< 3 vs. ≥ 3 lesions), T category (Ta vs. Tis/T1), concomitant CIS, and tumor grade (G1/2 vs. G3). Adjustments for re-TUR procedures, and separate analyses in patients who received it or not, compared the benefit in outcomes among the strains. Statistical analysis was performed Stata version 16.1 (Stata Corporation, College Station, TX, USA) with statistical significance set as p < 0.05.

## Results

422 patients with a median age of 67 (IQR: 64–70 were analyzed, 146 (34.6%) received Connaught, 112 (26.5%) received TICE and 164 (38.7%) received RIVM. Demographics and tumor characteristics are in Table 1. The three groups were equal in follow-up, age, gender, and other CUETO prognostic risk factors. We observed a significant difference in re-TUR within the groups with 30.1%, 48.2% and 56.7% for Connaught, TICE and RIVM respectively (*p* < 0.01). Median follow-up was 6.1 years with maximum of 14 years. Most were high-risk (*n = *392, 92.9%) with primary HG Ta-T1 the most common (*n = *216, 69.4%). Smaller proportions were seen of recurrent HG Ta-T1 (*n = *77, 18.2%), LG T1 (*n = *66, 15.6%), multiple large recurrent LG Ta (*n = *33, 7.8%) or those who had previously received intravesical chemotherapy for low-risk tumors (*n = *41; 9.7%). The median number of BCG instillations was 16 (IQR: 12–19), 19 (IQR: 15.75–21) and 20 (IQR: 16–21) for Connaught, TICE and RIVM, respectively (*p* < 0.01; Supplementary Fig. 2). Fewer Connaught instillations were due to a lower side effect tolerability and higher drop-off rate (Supplementary Table 1).

**Table 1:** Baseline characteristics of the study population according to BCG strain

	Connaught		TICE		RIVM		Total		*p* value
	*n = 146*	*%*	*n = 112*	*%*	*n = 164*	*%*	*n = 422*	*%*	
Median age (IQR)									0.168
	68 (65—70)		67 (62—69)		67 (63—69)		67 (64—69)		
Age, *n* (%)									0.052
< 60 year	11	7.5	18	16.1	16	9.8	45	10.7	
60–70 year	89	61	70	62.5	114	69.5	273	64.7	
≥ 70 year	46	31.5	24	21.4	34	20.7	104	24.6	
Gender									
Male	105	71.9	76	67,9	111	67,7	292	69,2	0.689
Female	41	56.1	36	32,1	53	32,3	130	30,8	
Smoking history									
Never	46	31,5	34	30,4	59	36,0	139	32,9	0.041
Previous	68	46,6	53	47,3	84	51,2	205	48,6	
Active	32	21,9	25	22,3	21	12,8	78	18,5	
Recurrence status									
Primary	99	67,8	71	63,4	107	65,2	277	65,6	0.761
Recurrent	47	32,2	41	36,6	57	34,8	145	34,4	
Previous intravesical CHT									0.731
Yes	14	9,6	9	8,0	18	11,0	41	9,7	
No	132	90,4	103	92,0	146	89,0	381	90,3	
re-TUR procedures									< 0.01
Yes	44	30,1	54	48,2	93	56,7	191	45,3	
No	102	69,9	58	51,8	71	43,3	231	54,7	
T stage									
Ta	54	37.0	32	28.6	42	25.6	128	30.3	0.292
Tis	9	6.2	8	7.1	13	7.9	30	7.1	
T1	83	56.8	72	64.3	109	66.5	264	62.6	
Tumor grade, WHO 1973									0.111
G3	104	71.2	89	79.5	129	78.7	322	76.3	
G2	17	11.6	5	4.5	7	4.3	29	6.9	
G1	25	17.1	18	16.1	28	17.1	71	16.8	
Tumor focality									0.141
< 3	55	37,7	31	27,7	63	38,4	149	35,3	
≥ 3	91	62,3	81	72,3	101	61,6	273	64,7	
Concomitant CIS									0.477
Yes	23	15,8	15	13,4	18	11,0	56	13,3	
No	123	84,2	97	86,6	146	89,0	366	86,7	
Median follow-up, (IQR)									0.079
	72 (55.2–91)		73 (69—85)		67 (60—85)		72 (60—85)		
EAU guidelines risk-group									0.846
Intermediate-risk	10	6,9	7	6,2	13	7,9	30	7,1	
High-risk	136	93,1	105	93,8	151	92,1	392	92,9	

**Fig. 1:**
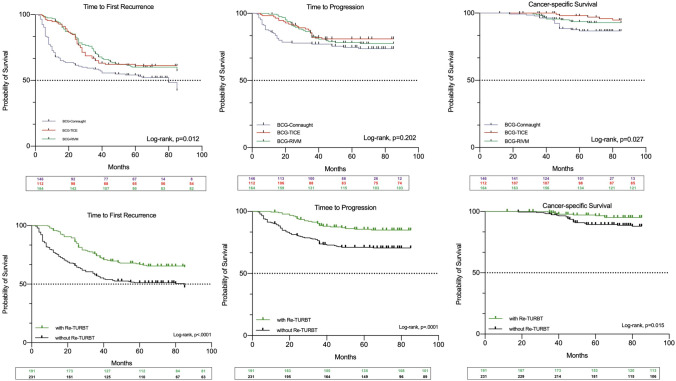
Kaplan–Meier curves (log-rank) depicting the univariate effect of BCG strains (**a**) and re-TUR (**b**) on survival outcomes

70 (47.9%) patients on Connaught, 43 (38.4%) on TICE and 68 on RIVM (41.5%) recurred (Supplementary Table 2a). Recurrence rate, stratified by re-TUR, was 43.8% on Connaught, 22.2% on TICE and 34.4% on RIVM. RFS at 5-year was 54.1% (95% CI 45.9–62.3), 61.3% (95% CI 52.2–70.3) and 60.2% (95% CI 52.6–67.7), respectively (log-rank, *p = *0.012; Dunn–Sidak: Connaught vs. TICE, *p = *0.052; Connaught vs. RIVM, *p = *0.029; TICE vs. RIVM, *p = *0.991) (Fig. 1a).

After adjustment for CUETO prognostic factors, patients who had undergone re-TUR had a prolonged time to first recurrence (hazard ratio [HR] 0.63; 95% CI 0.46–0.85). The difference was only significant in Connaught patients (HR 0.51; 95% CI 0.32–0.89) and TICE (HR 0.29, 95% CI 0.15–0.58) and not in RIVM (HR 0.81; 95% CI 0.47–1.38).

Patients who received TICE showed a longer time to first recurrence in comparison with Connaught (HR 0.52, 95% CI 0.35–0.77). This was also found comparing RIVM vs. Connaught (HR 0.55, 95% CI 0.39–0.78) but not between TICE and RIVM (HR 0.94, 95% CI 0.65–1.62) (Supplementary Table 3a). Similarly, the difference in time to first recurrence after CUETO risk factors adjustment and adding re-TUR, remained significant only for TICE and RIVM vs. Connaught (HR 0.55; 95% CI 0.37–0.81 and 0.58, 95% CI 0.41–0.82) (Supplementary Table 2a).

**Fig. 2:**
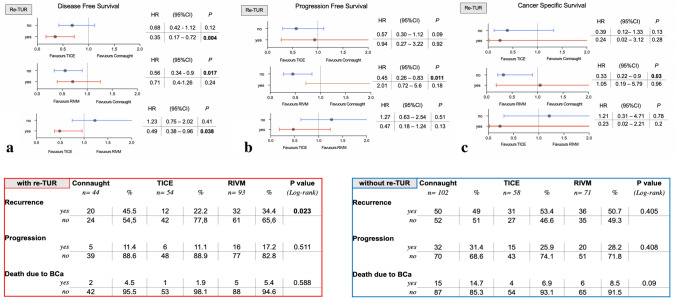
Sub-group analysis of stratified survival outcomes (**a** time to first recurrence, **b** time to progression; **c** cancer-specific survival) according to the presence or not of repeated trans-urethral resection (re-TUR)

In sub-analysis of only re-TUR patients (*n = *191), TICE was the sole strain to significantly prolong time to first recurrence compared to Connaught and RIVM (HR 0.35, 95% CI 0.17–0.72, and HR 0.49, 95% CI 0.38–0.96, respectively). The same was true only for RIVM compared to Connaught in the sub-analysis of patients who did not have re-TUR (*n = *231) (HR 0.56, 95% CI 0.34–0.98) (Fig. 2a).

37 (25.3%) patients on Connaught, 21 (18.7%) on TICE and 36 (22%) on RIVM progressed to MIBC (Supplementary Table 2b). Progression rate increased to 31.4% on Connaught, 25.9% on TICE and 28.2% on RIVM for those cases who did not undergo re-TUR. Progression rate was statistically equivalent for all three strains down to 11.4%, 11.1% and 17.2%, respectively, in cases of re-TUR. PFS at 5-yr was 74.7% (95% CI 67.5–81.9) for Connaught, 81% (95% CI 73.6–88.3) for TICE and 78.2% (95% CI 71.8–84.6) for RIVM, respectively (log-rank, *p = *0.202) (Fig. 1b).

Re-TUR before BCG also prolonged time to progression (HR 0.55; 95% CI 0.35–0.87). This was especially true in the group of patients who received Connaught and TICE (HR 0.27, 95% CI 0.19–0.72 and 0.37, 95% CI 0.14–0.98).

When adjusting for CUETO predictors, as well as for CUETO plus re-TUR, we did not find a statistically significant difference among the three strains in time to progression, but a trend towards significance was observed for TICE and RIVM compared to Connaught (HR 0.62, 95% CI 0.36–1.1 and 0.65, 95% CI 0.40–1.04 respectively; Supplementary Tables 2b–3b).

In the sub-analysis of re-TUR groups, when looking at only those who received it, no differences were demonstrated among all three stains for time to progression. When Re-TUR was not performed, RIVM was superior to Connaught (HR 0.45, 95% CI 0.26–0.83) (Fig. 2b).

Overall, 17 patients (11.6%) on Connaught, 5 (4.5%) on TICE and 11 (6.7%) on RIVM died due to BCa (Supplementary Table 2c). In total, 14.7%, 6.9% and 8.5% died with Connaught, TICE and RIVM when no re-TUR was performed and 4.5%, 1.9% and 5.4% with re-TUR. Five-year CSS was 86.7% (95% CI 80.8–92.6) for Connaught, 98% (95% CI 95.3–100) for Tice and 93.6% (95% CI 89.7–97.4) for RIVM (Log-rank: 0.027; Dunn–Sidak: Connaught vs. TICE, *p = *0.013; Connaught vs. RIVM, *p = *0.08; TICE vs. RIVM, *p = *0.42) (Fig. 1c).

Although a protective effect was noted, re-TUR was not independently associated with prolonged time to BCa death (HR 0.64, 95% CI 0.27–1.47). Similarly, for PFS none of the strains was independently associated with improved CSS (Supplementary Tables 2c–3c). RIVM again showed a protective effect in sub-group analysis of non-re-TUR patients (HR 0.33, 95% CI 0.22–0.94; Fig. 2c).

## Discussion

The genomic changes over 40–50 years which characterized several sub-strains may have attenuated potential efficacy (Gan et al. 2013). This may have led to imbalances in survival outcomes worldwide, especially in the BCG shortage era. Other trials compare strains (Huang et al. 2017), but study design and implementation of induction therapy alone led to mixed results in determining superiority. Of note, even though RIVM is the third most commonly used BCG strain worldwide (Gsponer et al. 2012), it remains the least studied.

We compared a large sample of patients treated with the three most representative BCG strains. To our knowledge, this is the first series with direct comparison of survival outcomes in these cohorts. Our patients were consistently treated with standardized maintenance. We also determined re-TUR’s importance on survival outcomes and the relative effect combined with specific strains.

The literature shows that re-TUR on high-risk and selected intermediate-risk NMIBCs improves outcomes (Krajewski et al. 2020). Eroglu et al. (2020) demonstrated the benefit of re-TUR on RFS and PFS and showed that re-TUR was an independent determinant of overall survival. We also found that re-TUR was independently associated with improvement in both recurrence and progression outcomes.

We found both TICE and RIVM superior to Connaught for prolonging DFS. Witjes et al. showed that maintenance TICE performs better than Connaught in HG T1 patients (Witjes et al. 2016). Connaught has a higher earlier immune response during induction but loses immune response efficacy during maintenance. Rentsch et al. (2014) showed Connaught conferred significantly greater 5-year RFS compared to TICE (*p = *0.0108) only with a sole induction course. Mice studies suggest that Connaught induced greater initial immune response than TICE. TICE seems to reach its optimum response over time, and therefore with maintenance shows longer DFS.

We found a similar advantage of RIVM over Connaught and a similar percentage of RFS for RIVM to what has previously been seen (Krajewski et al. 2018). Few other studies have reported RIVM’s comparative efficacy in NMIBC treatment. RIVM has been studied in comparison with intravesical chemotherapy or alone, not allowing for comparison (Vegt et al. 1995; Krajewski et al. 2018; Sengiku et al. 2013; Kaisary 1987). RIVM has demonstrated excellent RFS and PFS outcomes and 70% completion rate, suggesting good tolerability (Farah et al. 2014).

PFS was similar to that in the literature (Witjes et al. 2016; Nicolazzo et al. 2017 Oct; Nicolazzo et al. 2019; D'Andrea et al. 2020). No strain in our analysis was independently associated with longer PFS. We did observe a consistent trend towards and advantage of TICE and RIVM for PFS and CSS when compared to Connaught (Supplementary Tables 2b–2c).

BCG efficacies were almost overlapping after CUETO risk factors ± re-TUR adjustments, thus precluding definitive conclusions on the effect of re-TUR on strain performance in a real-life setting. But we observed two interesting outcomes regarding TICE and RIVM when our analyses were stratified according to the subgroups who had received re-TUR. TICE was the sole strain to improve RFS in re-TUR patients while RIVM provided longer PFS and CSS in the subgroup of patients who had not received re-TUR (Fig. 2a, b).

These findings corroborate that NMIBCs submitted to re-TUR followed by maintenance might achieve RFS with any of these three strains. But when no re-TUR is performed, our results suggest RIVM provides a potential intrinsic protection against MIBC progression and death from BCa which merits further evaluation. To our knowledge, this is the first series reporting a direct comparison among these strains in a maintenance setting. We cannot point to specific survival advantages, but our data suggest future avenues incorporating re-TUR.

A potential explanation for the consistently better performance of TICE and RIVM compared to Connaught may be better tolerability (Miyazaki et al. 2013). This was seen in our higher drop-off rate in the Connaught and was clearly associated with a median of fewer total instillations.

Our study has limitations, including the limitations of retrospective studies. This was not a randomly allocated head-to-head investigation. There may be selection biases between the groups that we could not account for. There was an imbalance between the groups who underwent re-TUR. Nevertheless, our findings are consistent with previous studies and use long-term follow-up to compare three different strains in the same study. Moreover, the strict eligibility criteria, strict BCG protocol homogeneity, and consistent maintenance allowed us to reliably explore the impact of re-TUR.

## Conclusions

Our study showed the RFS benefit of both TICE and RIVM compared to Connaught when administered with a maintenance protocol. We also corroborated the importance of performing routine re-TUR in intermediate-/high-risk NMIBCs. We did not find significant differences between TICE and RIVM for the analyzed survival outcomes. Stratifying our data for re-TUR revealed some benefits of TICE for RFS and RIVM for PFS end points. Future trials are needed on this topic in the BCG shortage era.

## Supplementary Information

Below is the link to the electronic supplementary material.

Supplementary Figure 1. Study design and treatment allocation for the three different BCG strains. NMIBC: non-muscle invasive bladder cancer; MIBC: muscle-invasive bladder cancer; TURBT: urethral resection of bladder tumor; BCG: bacillus Calmette-Guérin; BCa: bladder cancer (DOCX 566 KB)

Supplementary Figure 2. Overall number of instillations administered over the follow up according to the different BCG strain. P values according to one-way ANOVA (DOCX 265 KB)

Supplementary file3 (DOCX 19 KB)

Supplementary file4 (DOCX 20 KB)

Supplementary file5 (DOCX 17 KB)
