# Supplementary figures and images for: Efficacy of three BCG strains (Connaught, TICE and RIVM) with or without secondary resection (re-TUR) for intermediate/high-risk non-muscle-invasive bladder cancers: results from a retrospective single-institution cohort analysis
Source: J Cancer Res Clin Oncol. 2021 Mar 6;147(10):3073–80. doi: 10.1007/s00432-021-03571-0 (PMC8397662; doi:10.1007/s00432-021-03571-0)

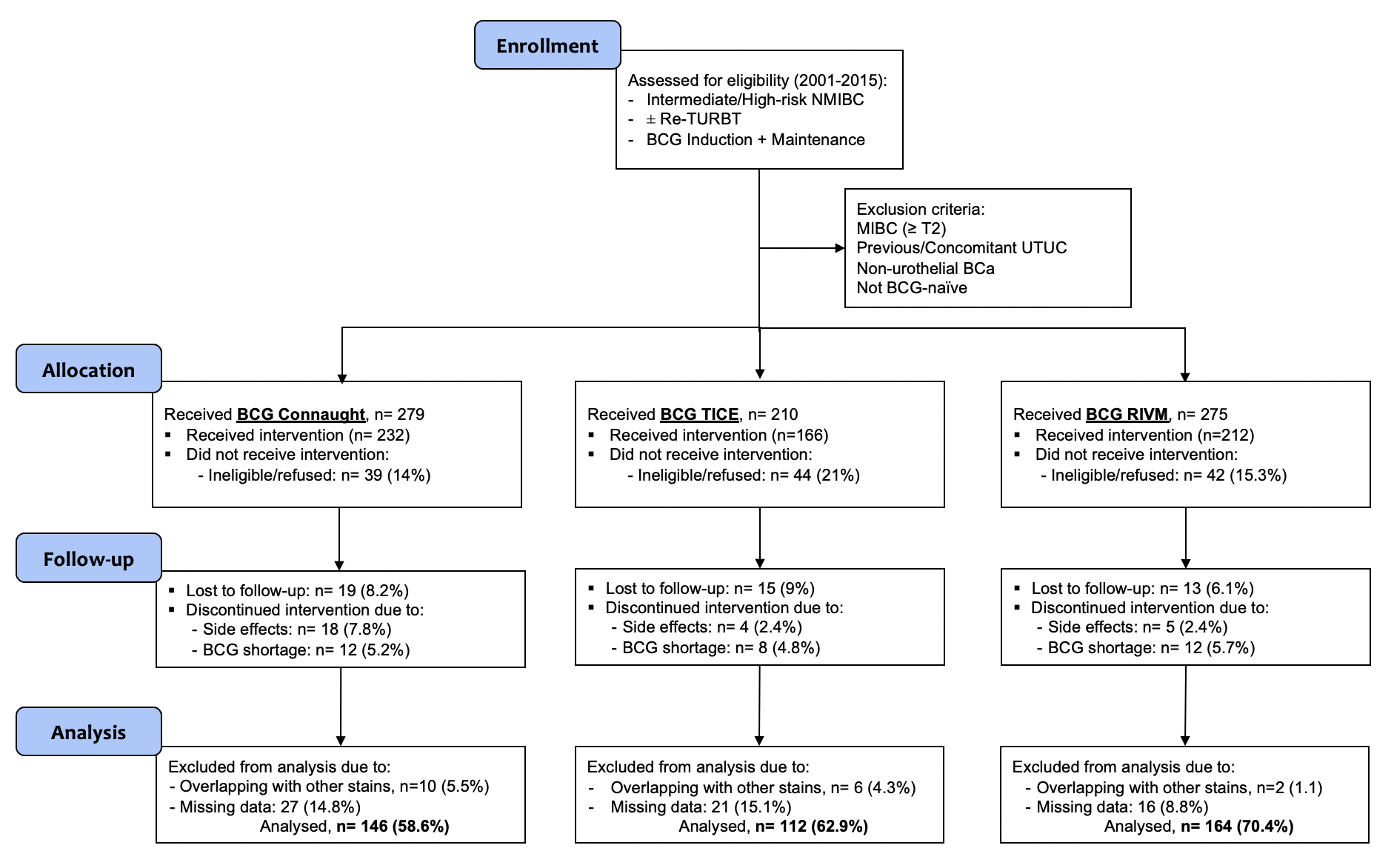

Supplement: Supplementary file 1 — Supplementary Figure 1. Study design and treatment allocation for the three different BCG strains. NMIBC: non-muscle invasive bladder cancer; MIBC: muscle-invasive bladder cancer; TURBT: urethral resection of bladder tumor; BCG: bacillus Calmette-Guérin; BCa: bladder cancer (DOCX 566 KB) [file 432_2021_3571_MOESM1_ESM.docx]

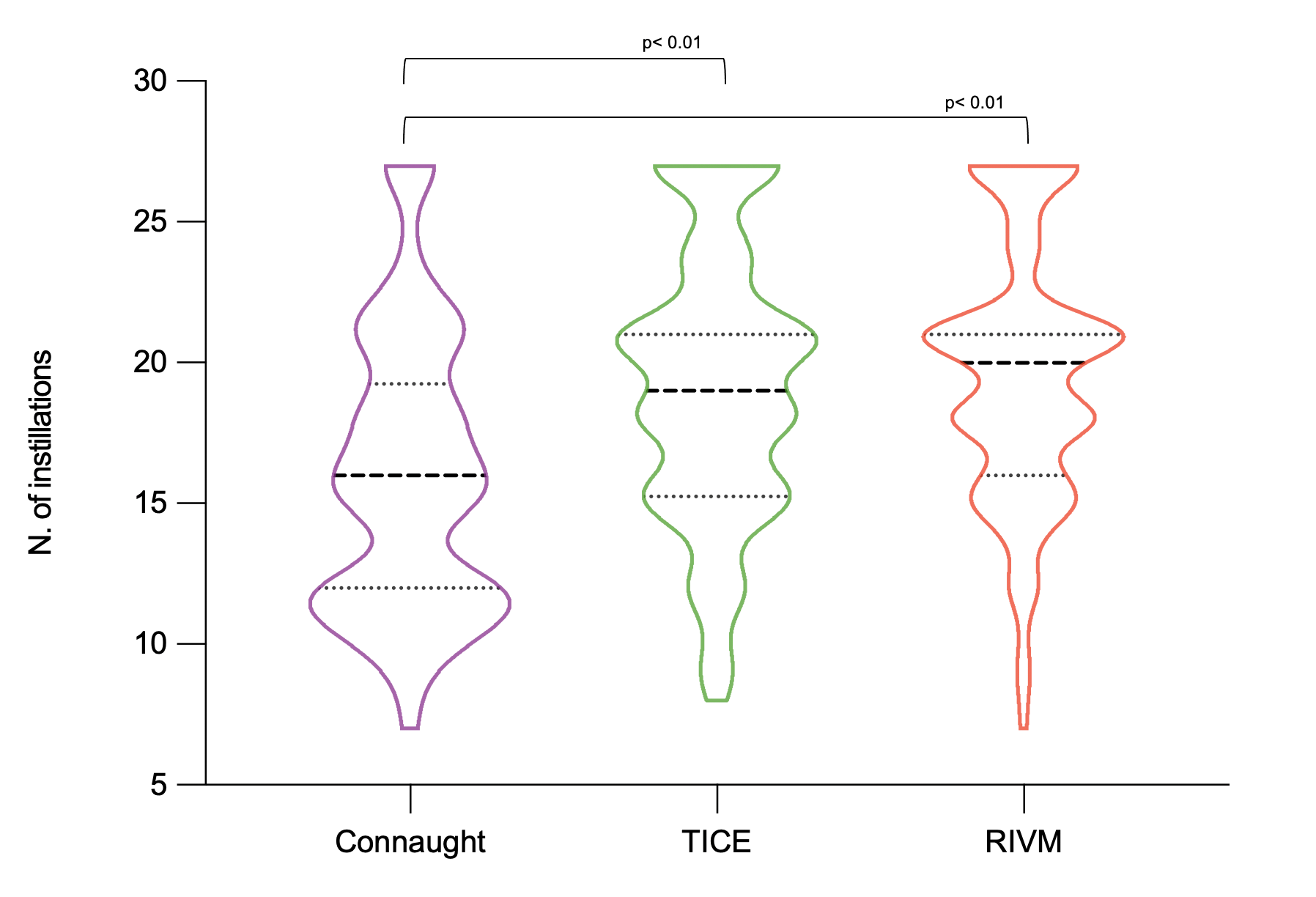

Supplement: Supplementary file 2 — Supplementary Figure 2. Overall number of instillations administered over the follow up according to the different BCG strain. P values according to one-way ANOVA (DOCX 265 KB) [file 432_2021_3571_MOESM2_ESM.docx]
