# Supplementary material for: Efficacy of three BCG strains (Connaught, TICE and RIVM) with or without secondary resection (re-TUR) for intermediate/high-risk non-muscle-invasive bladder cancers: results from a retrospective single-institution cohort analysis
Source: J Cancer Res Clin Oncol. 2021 Mar 6;147(10):3073–80. doi: 10.1007/s00432-021-03571-0 (PMC8397662; doi:10.1007/s00432-021-03571-0)
Supplement: Supplementary file 3 — Supplementary file3 (DOCX 19 KB) [file 432_2021_3571_MOESM3_ESM.docx]

| **Supplementary Table 1.** BCG schedule and tolerability according to the different strains and EAU risk groups | | | | | | | | | | | | | | |
| --- | --- | --- | --- | --- | --- | --- | --- | --- | --- | --- | --- | --- | --- | --- |
|  | **Connaught** (n=146) | | | |  | **TICE** (n=112) | | | |  | **RIVM** (n=164) | | | |
|  | **Intermediate-risk** | **%** | **High-risk** | **%** |  | **Intermediate-risk** | **%** | **High-risk** | **%** |  | **Intermediate-risk** | **%** | **High-risk** | **%** |
| **Number (%)** | *10* | *6.8* | *136* | *93.2* |  | *7* | *6.3* | *105* | *93.7* |  | *13* | *7.9* | *151* | *92.1* |
| **Number of instillations:** |  |  |  |  |  |  |  |  |  |  |  |  |  |  |
| *7–15* | 4 | 2.7 | 68 | 46.6 |  | 3 | 2,7 | 25 | 22,3 |  | 3 | 1,8 | 31 | 18,9 |
| *16-21* | 4 | 2.7 | 50 | 34.2 |  | 2 | 1,8 | 55 | 49,1 |  | 8 | 4,9 | 85 | 51,8 |
| *≥22* | 2 | 1.4 | 18 | 12.3 |  | 2 | 1,8 | 25 | 22,3 |  | 2 | 1,2 | 35 | 21,3 |
| **BCG dosage:** |  |  |  |  |  |  |  |  |  |  |  |  |  |  |
| *full dose* | 9 | 6.2 | 122 | 83.6 |  | 7 | 6,3 | 98 | 87,5 |  | 12 | 7,3 | 139 | 84,8 |
| *half dose* | 1 | 0.7 | 11 | 7.5 |  | 0 | 0,0 | 6 | 5,4 |  | 1 | 0,6 | 9 | 5,5 |
| *one-third* | 0 | 0,0 | 3 | 0.7 |  | 0 | 0,0 | 1 | 0,9 |  | 0 | 0,0 | 3 | 1,8 |
| **Duration of maintenance:** |  |  |  |  |  |  |  |  |  |  |  |  |  |  |
| *completed 3-yr schedule* | 2 | 1.4 | 7 | 4.8 |  | 1 | 0,9 | 16 | 14.3 |  | 2 | 1,2 | 21 | 12.8 |
| *completed 1 yr schedule* | 5 | 3.4 | 78 | 53.4 |  | 5 | 4,5 | 78 | 69.6 |  | 10 | 6,1 | 120 | 73.2 |
| *maintenance for < 1yr* | 3 | 2.1 | 51 | 34.9 |  | 1 | 0,9 | 11 | 9,8 |  | 1 | 0,6 | 10 | 6,1 |
| **Tolerability profile:** |  |  |  |  |  |  |  |  |  |  |  |  |  |  |
| *local toxicity* | 2 | 1.4 | 38 | 26 |  | 1 | 0,9 | 15 | 13,4 |  | 3 | 1,8 | 24 | 14,6 |
| *systemic toxicity* | 1 | 0.7 | 7 | 4.8 |  | 1 | 0,9 | 6 | 5,4 |  | 1 | 0,6 | 8 | 4,9 |
| *local and systemic toxicity* | 4 | 2.7 | 26 | 17.8 |  | 2 | 1,8 | 9 | 8,0 |  | 1 | 0,6 | 10 | 6,1 |
