# Supplementary material for: Efficacy of three BCG strains (Connaught, TICE and RIVM) with or without secondary resection (re-TUR) for intermediate/high-risk non-muscle-invasive bladder cancers: results from a retrospective single-institution cohort analysis
Source: J Cancer Res Clin Oncol. 2021 Mar 6;147(10):3073–80. doi: 10.1007/s00432-021-03571-0 (PMC8397662; doi:10.1007/s00432-021-03571-0)
Supplement: Supplementary file 4 — Supplementary file4 (DOCX 20 KB) [file 432_2021_3571_MOESM4_ESM.docx]

| **Supplementary Table 2.** Multivariable adjusted comparison of different BCG strains according to survival-specific prognostic factors and re-staging procedures | | | | | | | | | | | | |
| --- | --- | --- | --- | --- | --- | --- | --- | --- | --- | --- | --- | --- |
|  | **Connaught** |  | **TICE** |  | **RIVM** |  | P value |  |  |  |  |  |
|  | n= 146 | % | n= 112 | % | n= 164 | % | (Log-rank) |  | Multiple comparison* | HR | (95%CI) | P value |
| **Recurrence** | |  |  |  |  |  |  |  | **A** |  |  |  |
| yes | 70 | 47.9 | 43 | 38.4 | 68 | 41.5 | **0.012** |  | TICE vs. Connaught | 0.55 | 0.37 – 0.81 | **0.003** |
| no | 76 | 52.1 | 69 | 61.6 | 96 | 58.5 |  |  | RIVM vs. Connaught | 0.58 | 0.41 – 0.82 | **0.002** |
|  |  |  |  |  |  |  |  |  | TICE vs. RIVM | 0.95 | 0.64 – 1.40 | 0.79 |
| **Progression** | |  |  |  |  |  |  |  | **B** |  |  |  |
| yes | 37 | 25.3 | 21 | 18.8 | 34 | 14 | 0.202 |  | TICE vs. Connaught | 0.62 | 0.36 – 1.1 | 0.093 |
| no | 109 | 74.7 | 91 | 81.3 | 130 | 86 |  |  | RIVM vs. Connaught | 0.65 | 0.4 – 1.04 | 0.074 |
|  |  |  |  |  |  |  |  |  | TICE vs. RIVM | 0.97 | 0.56 – 1.67 | 0.89 |
| **Death due to BCa** | |  |  |  |  |  |  |  | **C** |  |  |  |
| yes | 17 | 11.6 | 5 | 4.5 | 11 | 6.7 | **0.027** |  | TICE vs. Connaught | 0.40 | 0.14 – 1.18 | 0.09 |
| no | 129 | 88.4 | 107 | 95.5 | 153 | 93.3 |  |  | RIVM vs. Connaught | 0.48 | 0.22 – 1.08 | 0.08 |
|  |  |  |  |  |  |  |  |  | TICE vs. RIVM | 0.82 | 0.28 – 2.43 | 0.73 |

*Cox hazard multivariable regression model adjusted for CUETO risk factors (age, gender, tumor focality, T stage, Grade, recurrence status).
