# Supplementary material for: Efficacy of three BCG strains (Connaught, TICE and RIVM) with or without secondary resection (re-TUR) for intermediate/high-risk non-muscle-invasive bladder cancers: results from a retrospective single-institution cohort analysis
Source: J Cancer Res Clin Oncol. 2021 Mar 6;147(10):3073–80. doi: 10.1007/s00432-021-03571-0 (PMC8397662; doi:10.1007/s00432-021-03571-0)
Supplement: Supplementary file 5 — Supplementary file5 (DOCX 17 KB) [file 432_2021_3571_MOESM5_ESM.docx]

| **Supplementary table 3**. Multivariable adjusted comparison of the different BCG strains according to survival-specific prognostic factors | | | | | | | | | | | | |
| --- | --- | --- | --- | --- | --- | --- | --- | --- | --- | --- | --- | --- |
|  | **Connaught** |  | **TICE** |  | **RIVM** |  | **P value** |  |  |  |  |  |
|  | n= 146 | % | n= 112 | % | n= 164 | % | (Log-rank) |  | Multiple comparison* | **HR** | **(95%CI)** | **P value** |
| **Recurrence** | |  |  |  |  |  |  |  | **A** |  |  |  |
| yes | 70 | 47.9 | 43 | 38.4 | 68 | 41,5 | **0.012** |  | TICE vs. Connaught | 0.52 | 0.35 – 0.77 | **0.001** |
| no | 76 | 52.1 | 69 | 61.6 | 96 | 58,5 |  |  | RIVM vs. Connaught | 0.55 | 0.39 – 0.78 | **0.001** |
|  |  |  |  |  |  |  |  |  | TICE vs. RIVM | 0.94 | 0.64 – 1.38 | 0.75 |
| **Progression** | |  |  |  |  |  |  |  | **B** |  |  |  |
| yes | 37 | 25.3 | 21 | 18.8 | 34 | 20.7 | 0.202 |  | TICE vs. Connaught | 0.58 | 0.34 – 1.03 | 0.056 |
| no | 109 | 74.7 | 91 | 81.3 | 130 | 79.3 |  |  | RIVM vs. Connaught | 0.62 | 0.39 – 0.99 | **0.045** |
|  |  |  |  |  |  |  |  |  | TICE vs. RIVM | 0.94 | 0.55 – 1.62 | 0.83 |
| **Death due to BCa** | |  |  |  |  |  |  |  | **C** |  |  |  |
| yes | 17 | 11.6 | 5 | 4.5 | 11 | 6.7 | **0.027** |  | TICE vs. Connaught | 0.39 | 0.13 – 1.09 | 0.059 |
| no | 129 | 88.4 | 107 | 95.5 | 153 | 93.3 |  |  | RIVM vs. Connaught | 0.44 | xxx – 0.96 | **0.04** |
|  |  |  |  |  |  |  |  |  | TICE vs. RIVM | 0.82 | 0.28 – 2.39 | 0.71 |
